# Prediction of Sperm Progression in Three Dimensions Using Rapid Optical Imaging and Dynamic Mechanical Modeling

**DOI:** 10.3390/cells11081319

**Published:** 2022-04-13

**Authors:** Mayssam Nassir, Mattan Levi, Gili Dardikman-Yoffe, Simcha K. Mirsky, Natan T. Shaked

**Affiliations:** Department of Biomedical Engineering, Faculty of Engineering, Tel Aviv University, Tel Aviv 69978, Israel; mayssam.nassir@gmail.com (M.N.); mattanlevi@gmail.com (M.L.); gilidard@gmail.com (G.D.-Y.); simcha.mirsky@gmail.com (S.K.M.)

**Keywords:** sperm, interferometric computed tomography, tomographic phase microscopy, biomechanical modeling

## Abstract

We present a multidisciplinary approach for predicting how sperm cells with various morphologies swim in three-dimensions (3D), from milliseconds to much longer time scales at spatial resolutions of less than half a micron. We created the sperm 3D geometry and built a numerical mechanical model using the experimentally acquired dynamic 3D refractive-index profiles of sperm cells swimming in vitro as imaged by high-resolution optical diffraction tomography. By controlling parameters in the model, such as the size and shape of the sperm head and tail, we can then predict how different sperm cells, normal or abnormal, would swim in 3D, in the short or long term. We quantified various 3D structural factor effects on the sperm long-term motility. We found that some abnormal sperm cells swim faster than normal sperm cells, in contrast to the commonly used sperm selection assumption during in vitro fertilization (IVF), according to which sperm cells should mainly be chosen based on their progressive motion. We thus establish a new tool for sperm analysis and male-infertility diagnosis, as well as sperm selection criteria for fertility treatments.

## 1. Introduction

Human sperm cell morphology and motility are major factors in the diagnosis and prognosis of male fertility, as well as for sperm selection for in vitro fertilization (IVF), where the male sperm cells fertilize the female egg in a dish. Most sperm cells that reach the fertilization site in the female body have normal morphology according to WHO-2010. Normal morphology also correlates to some extent with other sperm potency parameters such as hyaluronic acid-binding, Zona-hamster assay, and a low DNA fragmentation index [1]. The physiological mechanisms of the female body for a natural selection of sperm cells are bypassed in IVF, and it is not possible to predict which individual sperm cell would be the one that is most likely to fertilize the egg naturally and result in a healthy child. Furthermore, it is not completely clear why normal morphology sperm cells are also good swimmers and the ones that would reach the fertilization site in the female body. Predicting the dynamics by which a human sperm cell moves in 3D relative to an ambient flow and various obstacles in the female body, for long distances, during natural fertilization, is very challenging. The biological mechanisms that connect sperm movement, morphology, contents, fertilization potential, and normal pregnancy are not completely understood. Previously, microscopic analysis together with theoretical modeling was used to illustrate and interpret the swimming strategies of sperm cells under complex flow conditions [2,3,4,5,6]. Tracking of human sperm cells across large volumes by digital imaging and analysis techniques has also been demonstrated [7,8,9,10]. Specifically, Su, et al. [9] used lens-free imaging on a chip, tracked the 3D trajectories of individual human sperm cells, and concluded that typically the sperm head moves forward swiftly along a slightly curved axis with a small lateral displacement. Dardikman-Yoffe, et al. [11] presented a quantitative optical microscopy method and reconstruction algorithms to provide highly detailed dynamic 3D refractive-index distributions of the sperm cell during the swim, including both the head with its internal-organelle morphologies and the flagellum.

The sperm flagellum has a detailed internal structure, the axoneme, which is powered by dynein molecular motors distributed regularly along its length and circumference. Dynein motors produce local force, which is translated into the controlled, regular beating of the global structure [12]. Considerable interest has been directed towards understanding the dynein activities by the development of a mechanical model that includes the force generation and activation dynamics of the dynein molecular motors [13,14]. The sperm flagellar axoneme consists of a central pair of singlet microtubules surrounded by nine outer doublet microtubules (9 + 2 structure) and encased by the cell membrane [15]. Radial links connect the central microtubule pair to each surrounding microtubule doublets, and nexin bridges connect adjacent doublets. Active and passive sliding between pairs of outer doublets by the dynein molecular motors induces shear forces and bending.

Classical models for ciliary and flagellar motion have been developed [6,7,16,17,18,19]. Advanced models that analyze in detail the axonemal structure and dynein activation, to explore and describe the internal ciliary and flagellar dynamics, have also been presented [20,21,22,23]. Most studies developed approximate models containing mechanical parts, which represent the main organs of the sperm cell. These models only partially describe the geometrical structure of sperm cells and consequently lead to a lack of understanding of the mechanical behavior of the sperm cell swimming. Previous models described the two-dimensional axonemal structure of planar beat patterns by two elastic rods separated by a fixed distance, linked by elastic structural elements and by active force generators, corresponding to the dynein motor proteins [24,25,26,27]. Specifically, recent research modeled sperm motility in a hydrodynamic simulation to study the broad deflection-angle distribution of sperm cells in zigzag channels [27]. For this purpose, they implemented a minimalistic sperm model, which consisted of two successional segments connected by nodes, with each node containing four beads connected by springs. Another study quantified the flagellar beats of sperm cells and determined the average time of the beating shape, as well as the first temporal Fourier mode of the beat patterns for different experimental conditions using a two-dimensional representation of the axoneme, in which two flexible filaments slid relative to each other in the beat plane [24]. A recent study found significant differences between fertile and non-fertile men with testicular germ cell tumors, with significant values of tail and head abnormalities [28].

In the current paper, we propose a multi-modal method that can predict how normal and pathological human sperm cells would swim in 3D based on multiple kinetic parameters describing the mechanical and physical dynamic behavior of sperm cells in 3D. We previously acquired dynamic 3D profiles of swimming normal human sperm cells using optical diffraction tomography, a stain-free quantitative optical imaging method [11]. Based on the reconstructed dynamic 3D profiles of the normal cells, we developed mechanical computational sperm-cell models, for both normal and abnormal sperm morphologies. These models describe the full sperm geometry with all cell organelles (head, flagellum, and midpiece), including all the internal components and structures for each organelle. We then reconstructed the mechanical behavior of each model by activating the dynein motors to describe a normal cell model behavior and compared it to an abnormal cell model behavior by changing various parameters, such as flagellum amplitude, wavelength, beat frequency, total and axial displacements and linear and angular velocities. Using these models, we can also evaluate the 3D swimming pattern of the sperm cells for much longer periods than can be acquired optically. Thus, this approach offers information that cannot be obtained using the previous methods. It provides new tools for evaluating the potential fertility of individual human sperm cells in reaching the egg, as well as a novel means for studying the interplay between sperm morphology and dynamics in 3D.

## 2. Materials and Methods

We built a numerical mechanical sperm model based on previous imaging experimental results of sperm cells done by our group [11]. A normal-morphology sperm cell swimming in fluid was acquired with our dynamic interferometric computer tomography method at spatial resolution of 0.5 microns, achieving both retrieval of the 3D refractive-index profile of the sperm head and the detailed 4D localization of the thin, highly dynamic sperm flagellum [11].

We used the natural head rotation of the sperm cell to acquire perspective projections of the head during swimming in a dish, without using any cell staining or special sample preparations [11]. Each projection was acquired using our clinic-ready off-axis interferometric module connected to the exit of a simple clinical inverted microscope. This optical setup acquires the sperm-cell projection wavefronts at 2000 frames per second. All projections are then processed to reconstruct the sperm 3D refractive-index profile via optical diffraction tomography, allowing us to dynamically image the sperm cell in 3D during swimming without using cell staining. The sperm flagellum and midpiece are reconstructed via holography, which enables numerical refocusing of points in thin and sparse structures, thus enabling the reconstruction of the 3D shape of the flagellum and midpiece in a single camera exposure, providing a method to track them at high 3D spatial and temporal resolutions. Due to the high temporal resolution required, we used a fast camera with an internal buffer, which limits the practical acquisition duration to no more than one second, preventing the prediction of the 3D swimming of sperm for longer periods.

Based on the experimental 3D swimming of the normal-morphology sperm cell, we developed 15 detailed dynamic 3D sperm cell models: one for a normal sperm cell and 14 for abnormal morphology sperm cells with different types of abnormalities. These include common defects in head and flagellum morphology. The most common sperm abnormalities, as defined in the 2010 World Health Organization (WHO-2010) guidelines [1], are observed as a tapered or thin head, a thin midpiece, and no tail or a short tail [28].

The models possess full sperm geometry with all sperm cell real organs (head, flagellum, and midpiece) including all internal components. The head model contains the nucleus, acrosome, and membrane. The midpiece (neck) is composed of the centrosome and mitochondria, covered by a membrane. The flagellum is constructed from nine doublet microtubules, a central double microtubule, radial spoke triplet, nexin links, and dynein motors and covered by the membrane. We used these models to simulate active and passive sliding between pairs of outer doublets by the dynein molecular motors inducing shear forces and bending of the axoneme. By activating the dynein motors in our full mechanical model, we can mathematically describe the mechanical behavior of each model over time, including the path and trend of motion, velocity, momentum, and beating pattern characteristics. These models can be used to predict how normal and pathological sperm cells would swim in 3D for durations that are much longer than can be acquired experimentally.

Normal sperm model: The model, possessing full sperm geometry including the head and tail with all internal components, is based on the experimentally acquired sperm cells obtained in a previous study [11]. The overall length of the tail is 55 µm, its diameter decreases from 1 µm to 0.1 µm at the distal end, and it is covered by a membrane. The tail is structurally divided into three major parts: the midpiece of length 4 µm and diameter of 1 µm, the principal piece of length 46 µm and diameter 0.5 µm, and the terminal segment of length 5 µm and diameter decreasing from 0.5 µm to 0.1 µm at the end. Supplementary Figure S1 shows the structural comparison between the optical and geometrical models, including the head and the flagellum of the normal sperm model.

**Figure 1:**
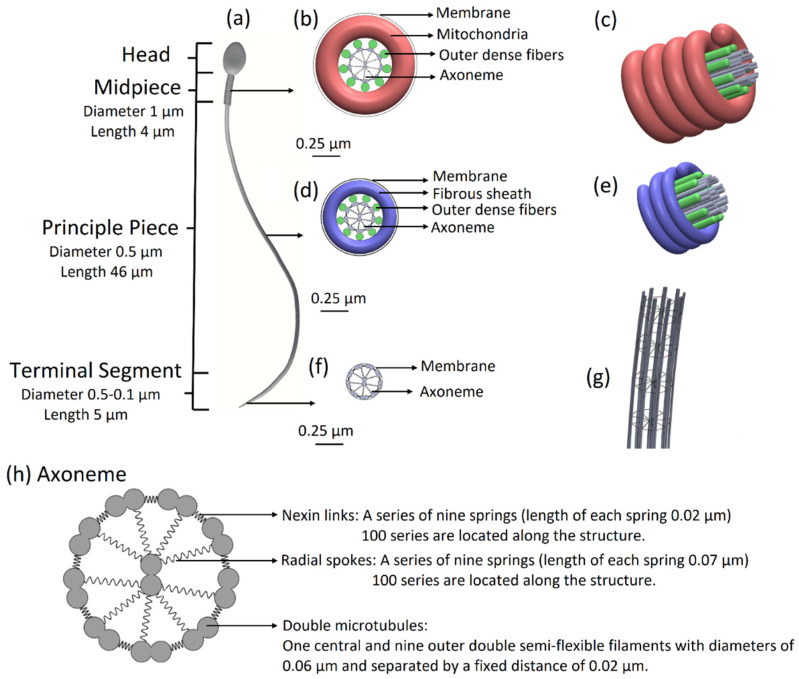
Sperm cell geometrical model. (**a**) Sperm model with all parts including the head, midpiece, principal piece, and terminal segment. (**b**) Midpiece in cross-section: axonemal structure surrounded by nine outer dense fibers and doubled spiral arrays of mitochondria. The midpiece is covered by a membrane. (**c**) 3D view of the midpiece. (**d**) The principal piece in cross-section: axonemal structure surrounded by nine outer dense fibers and fibrous sheath spiral and covered by a membrane. (**e**) 3D view of the principle piece. (**f**) The terminal segment in cross-section: axonemal structure covered by a membrane. (**g**) The 3D architecture of the flagellar structure model. (**h**) Axoneme in cross-section: axonemal structure: nine outer double semi-flexible filaments (microtubule doublets), linked by stiff springs (nexin links) and radial springs (radial spokes), connecting each outer filament with the central double semi-flexible filaments and covered by a membrane.

**Figure 2:**
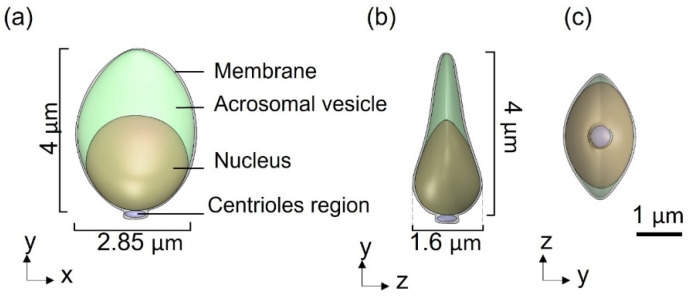
3D sperm head geometrical model: (**a**) Side view of the head model, containing the acrosomal vesicle, nucleus, membrane, and centrioles region. (**b**,**c**) Top and rear views of the head model.

Based on the experimental 3D swimming normal-morphology sperm cell [11], we built a 3D mechanical model of a sperm cell. The internal structure of the tail is comprised of a 3D axonemal structure of beat patterns with nine outer double semi-flexible filaments with diameters of 0.05 µm and separated by a fixed distance of 0.02 µm, linked by stiff springs corresponding to nexin links. Radial springs, presenting radial spokes, connect each outer filament with the central double semi-flexible filaments to stabilize the structure. Dynein motor proteins, represented by *N* = 100 nodes, are located along each outer double filament. The 3D axonemal structure is covered by an elastic membrane along the entire tail. The axonemal structure in the principle piece is surrounded by nine outer dense fibers and a fibrous sheath spiral, covered by a membrane. The midpiece contains an axonemal structure, surrounded by nine outer dense fibers and spiral arrays of mitochondria, and is covered by a membrane (Figure 1). The head of the model is approximated by an elliptical shape, 4.2 µm long and 2.85 µm wide with radial symmetrical tapering reaching the tip of the head, approximated as 0.46 µm. The head model contains the nucleus (56.75%), acrosome (28.4%), and membrane (14.86%) and is connected to the tail by the midpiece (Figure 2). All models were built in SolidWorks (Premium 2017 ×64 Edition, Waltham, MA, USA).

For verification, we performed a structural similarity comparison between the optical and geometrical models using the structural similarity image index (SSIM), which resulted in values of 91.1% for the sperm head and 89.7% for the sperm tail. Supplementary Figures S2 and S3 display the local SSIM maps, including the global SSIM values. High values of global SSIM indicate high correlation and similarity between the models.

**Figure 3:**
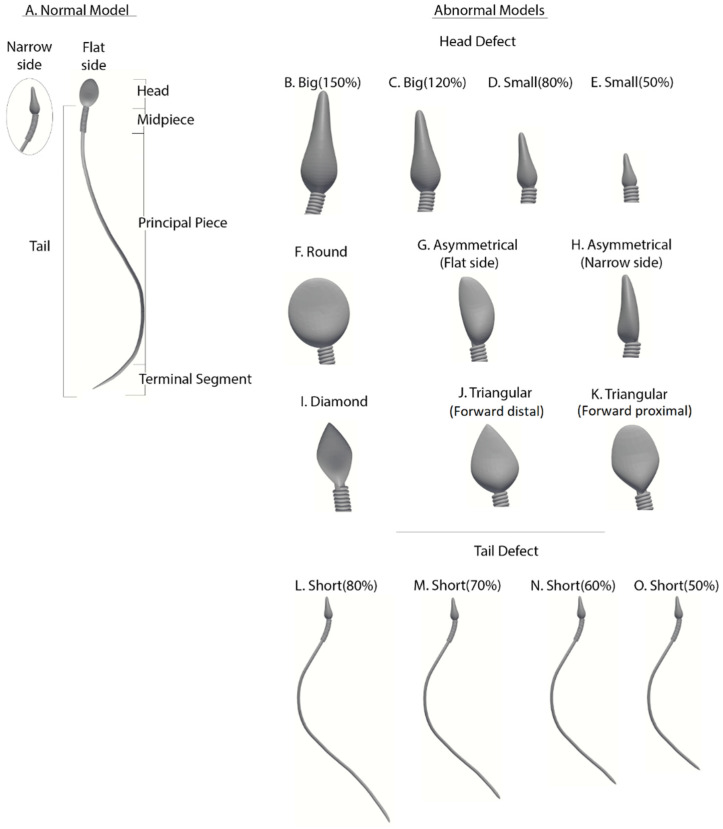
Normal and abnormal sperm cell models (according to the WHO classification). (**A**) Normal sperm cell model. (**B**–**K**) Different morphological abnormalities among sperm cells, (**B**–**E**) abnormalities in head size, (**F**–**K**) abnormalities in head shape, and (**L**–**O**) abnormalities in tail length.

Abnormal sperm models: Morphologically abnormal sperm models (Figure 3) are divided into three subgroups according to the defect category of the head or tail, based on real-world sperm pathologies according to the WHO-2010 guidelines.

- Abnormalities of head size: Four models (B–E) have abnormal head sizes (smaller or larger than normal), with the tail structure and head shape as normal. The two large-headed models are 150% (model B) and 120% (model C) of the normal head size. The two small-headed models are 80% (model D) and 50% (model E) of the normal head size.
- Abnormalities of head shape: Six models (F–K) have defects in the head shape and size, with the tail structure as normal. The head defects are expressed as follows: round head (model F), asymmetrical head on the flat side (model G), asymmetrical head on the narrow side (model H), diamond head (model I), tapered head on the forward side (model J), and tapered head on the midpiece side (model K).
- Abnormalities of the tail length: Four models (L–O) have short tails, with head morphology as normal. Tail lengths of models L, M, N, and O are 80%, 70%, 60%, and 50%, respectively, of the tail length of the normal model.

## 3. Beat Pattern

The developed model is a mechanical, mathematical model, based on kinetic laws and finite elements’ motion in 3D. Each element of the model is described as a node in a mesh and treated as an independent body, based on the finite-elements theory. Forces were applied to the elements, each of which moved in the 3D space creating its 3D path. The composition of all elements described the path of the cell.

Each dynein component was designated to be an element, sliding based on its curvature and position change. The inputs of our model are forces applied to the dynein components based on the “switch-point” principle, whereas fixed forces describe the external influential forces such as hydrodynamics and gradients. Specifically, we describe hydrodynamics as a fixed shear force that limits the element moves along the progress axis. Using Frenet equations, we converted the applied forces to position and rotation changes for each element. Finally, we used the location and rotation changes to create a simulation of the beating pattern of the sperm cell.

For this purpose, the three-dimensional axoneme is divided into two groups, three and six filaments, which are located on opposing sides [29]. The axonemal beating is created by activating the dynein motors in two filaments groups intermittently based on the “switch-point” principle [29,30,31,32]. This principle specifies that the flagellar beat pattern is caused by the periodic switching of spatially restricted, asymmetrical dynein activation in the two filament groups. In other words, axonemal bending in one direction is generated by activating the dynein motors in the same direction, while the dynein motors located along the second filament group on the opposite side are passive. Therefore, the action of dynein motors at opposite sides of the axoneme is superimposed in an antagonistic manner by switching flagellar sides relative to the bending direction. The number of active dynein motors along each filament is determined to be N=100 nodes. The beating patterns of the different models are unaffected by larger numbers of nodes but influence the simulation runtime and memory. Low simulation resolutions are obtained with fewer than 100 nodes of dynein motors.

We simulated the dynein sliding forces by a mathematical model based on position changes. We describe the shape of a filament by its curvature, r(s,t), where s is the dynein component. Each elastic filament can bend along the normal $\left(\hat{i}\right)$ and radial $\left(\hat{j}\right)$ directions and twist along the tangential direction $\left(\hat{k}\right)$ [33]. The change in the 3D positions along the curve of each filament is given by the Frenet equations:

(1) $$\frac{dT}{ds}=\kappa T,$$

(2) $$\frac{dN}{ds}=\tau B-\kappa T,$$

(3) $$\frac{dB}{ds}=-\tau N,$$

where κ is the curvature, and τ is the torsion of the curve. The tangent (T), normal (N), and binormal (B=T×N) unit vectors are the Frenet–Serret frame. Hence, the curvature *κ* and torsion *τ* of the curve is described by:

(4) $$\kappa =\left|\frac{dT\left(s\right)}{ds}\right|,$$

(5) $$\tau =\frac{1}{\kappa }\frac{dT}{ds}\frac{dB}{ds}.$$

The following equations define the twist degree by the intrinsic twist (φ) with respect to the Frenet frame:

(6) $$N\left(s\right)=\cos\varphi \hat{i}-\sin\varphi \hat{j},$$

(7) $$B\left(s\right)=\sin\varphi \hat{i}+\cos\varphi \hat{j},$$

(8) T(s)=k(s).

The change in the coordinate axis as a function of time is defined by the angular velocity vector of the Frenet frame of the curve (ω). This angular velocity in each segment is the combination of the rotations of each of the three Frenet vectors: $\omega =\omega_{T}+\omega_{N}+\omega_{B}$. Infinitesimally small changes of the orientation related to rotations around the three axes are:

(9) $$\partial_{s}\left(\begin{array}{c} i\left(s\right)\\ \begin{array}{c} j\left(s\right)\\ k\left(s\right) \end{array} \end{array}\right)=\left(\begin{array}{ccc} 0 & \omega_{T}\left(s\right) & -\omega_{B}\left(s\right)\\ -\omega_{T}\left(s\right) & 0 & \omega_{N}\left(s\right)\\ \omega_{B}\left(s\right) & -\omega_{N}\left(s\right) & 0 \end{array}\right)\left(\begin{array}{c} i\left(s\right)\\ \begin{array}{c} j\left(s\right)\\ k\left(s\right) \end{array} \end{array}\right).$$

Following the above equations, the curvature (κ) and torsion (τ) of the curve are given by:

(10) $$\kappa =\left|\frac{dT\left(s\right)}{ds}\right|=\sqrt{{\left(\omega_{N}\right)}^{2}+{\left(\omega_{B}\right)}^{2}},$$

(11) $$\tau =\frac{1}{\kappa }\frac{dT}{ds}\frac{dB}{ds}=\omega_{T}-\varphi .$$

By inverting Equation (11), and using the intrinsic twist (φ), the local curvatures in each dynein node along the active filament are:

(12) $$\omega_{N}\left(s,t\right)=\kappa \left(s,t\right)\sin\varphi \left(s,t\right),$$

(13) $$\omega_{B}\left(s,t\right)=\kappa \left(s,t\right)\cos\varphi \left(s,t\right),$$

(14) $$\omega_{T}\left(s,t\right)=\tau \left(s,t\right)+\frac{d\varphi }{ds}\left(s,t\right).$$

The curvature κ(s,t) of the filament is given by [34]:

(15) $$\kappa \left(s,t\right)=s^{2}\sin{\left(\frac{\pi }{3}\right)}^{-1}\times \delta_{N}\left(s,t\right),$$

where

(16) $$\delta_{N}\left(s,t\right)=A\sin\left(\frac{2\pi }{\lambda }s-\omega t\right).$$

The shape of a filament by its curvature, κ(s,t), is a function of the dynein component ‘s’ and time ‘t’ [34]. $\delta_{N}\left(s,t\right)$ is a step function (or staircase function), which defines the switch point mechanism and directly influences the model curvature. In the case of inactive filaments $\delta_{N}\left(s,t\right)=0$, no force will be applied on the elements, and the curvature of the specific element will not change. In the case of active filaments $\delta_{N}\left(s,t\right)\;\neq \;0$, the force will be applied on the elements and the curvature will be updated [15].

The amplitude $\left(A=0.63L_{flagellum\_length}\right)$, the wavelength $\left(\lambda =0.05r_{head\_radius}\right)$, and the frequency (ω=0.05 Hz) are the mechanical properties of the stiff springs [24,27]. The equations in the dynein motors along the inactive filaments are $\delta_{N}=\kappa \left(s,t\right)=\omega_{N,B,T}=0$.

## 4. Dynamic Simulation

To perform dynamic simulations, we meshed the 3D geometrical sperm models. We used the generation and processing toolbox by SolidWorks to create a 3D tetrahedral element mesh for this purpose. The mesh densities of the different models vary between the cells. This variability can affect the simulation runtime but does not change the outcome measures (less than 3%). The models were assumed to be hyper-elastic uncompressible materials with the Neo-Hookean constitutive model. The models were assumed to be uncompressible material with uniform mechanical proprieties for all components, such as the density. Since the cell head is the major component by weight, changing the density of the components is not expected to significantly affect the swimming behavior. We first analyzed the beating patterns of the sperm models when their heads were clamped to a fixed surface. The head was fixed for any progression and was free for 3D rotations. Secondly, we studied the sperm swimming inside an infinite Newtonian fluid when the head was free to swim. This Newtonian fluid is considered saline water with a viscosity of (1.02 mPa×s) at room temperature (25 °C). We assumed that there was no cell–surface interaction, that is, no-slip at the sidewalls, since the changes of the axonemal shape were caused by the internal force generators and were unaffected by the hydrodynamics. In addition, since the simulation still ensured low Reynold’s hydrodynamics, the qualitative description still held. The dynamic simulations were performed using Blender 2.91 and Python 3.3 SW (3D modeling and rendering package).

## 5. Outcome Measurements

The beating patterns of the different models were analyzed for position maps, calculated by Blender 2.91 Python 3.3 SW (3D modeling and rendering package). The localized displacements that developed in the dynein nodes, heads, and the flagellar axoneme of the normal and pathological models were determined as a function of the time. Outcome measures included:

(1) Flagellar beatings of the normal and pathological models with clamped heads.
(2) The 3D trajectories of the sperm head centroid of the normal and pathological models, across infinite fluid for 17 s.
(3) The kinetic and physical parameters of the three-dimensional swimming patterns, such as:
  - Distance (‘*x*’ axis): the axial displacement of the head centroid path in the forward direction.
  - Flagellum amplitude, calculated based on snapshots of the beating pattern that projected on the forward plane ‘*x* − *y*’.
  - Frequency f=1/T, where T is the time taken to complete one cycle of the beating pattern.
  - Linear velocity v=x/t, where x is the distance (*x* axis) in the forward direction as a function of time t.
  - Wavelength λ=v/f, where v is the linear velocity and f is the frequency.
  - Angular velocity w=v/r, where v is the linear velocity and r is the radius of the motion in the lateral plane ‘*z* − *y*’.
  - Momentum $P_{x,y,z}=m\times v_{x,y,z}$, where m is the model mass and $v_{x,y,z}$ is its axial velocity.
(4) Prediction for the normal and pathological model swimming patterns over 15 min using 3D polynomial regression.
(5) Scoring the various sperm cell pathologies according to their estimated success rates of reaching the egg.

## 6. Results

We distinguished between two simulation conditions: first, the case in which the sperm head was clamped (held at its tip) in a way that it could not move forward; second, the case in which the head was free to swim in the 3D space. In the case of the clamped head, we quantified the flagellar beats of the sperm models with high spatio–temporal resolution and determined the frequency and shape amplitude for each model.

### 6.1. Flagellar Beating of the Normal Sperm Model

**Figure 4:**
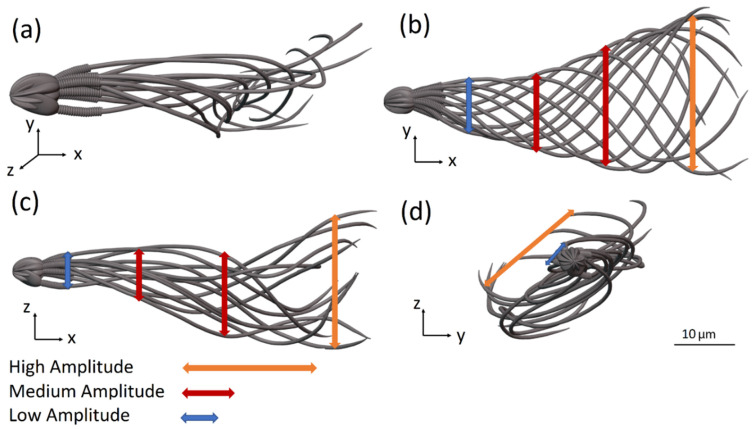
Flagellar beating of a normal sperm model with clamped head (held at its tip). (**a**) Wave form of the 3D flagellar beating. (**b**–**d**) The projections of the 3D flagellar beat in three orthogonal planes.

Figure 4a–d show 12 frames from 3D and 2D views of the flagellar beating of the swimming normal sperm model when the head was clamped by a solid boundary. The flagellum rolled counterclockwise around its swimming axis and each flagellar point rolled in helical trajectories. Most of sperm cells rotated counterclockwise around their swimming axis as reported in the literature [8,35,36]. We performed dynamic simulations with sperm cell rotation clockwise and counterclockwise to estimate the effect of the rotation direction changes on the cell behavior. The changes in the swimming paths were negligible; therefore, we assumed that the cell can rotate in one direction, counterclockwise. The wave form of the 3D flagellar beating is described in Figure 4a–c show the projections of the 3D flagellar beat in 2D. In Figure 4b,c, the flagellar waveform presents looping patterns with polar symmetry on both planes ‘*x* − *y*’ and ‘*x* − *z*’, while in Figure 4d, the wave form presents asymmetrical behavior in the frontal plane ‘*y* − *z*’, with curvature towards the tip [37].

**Figure 5:**
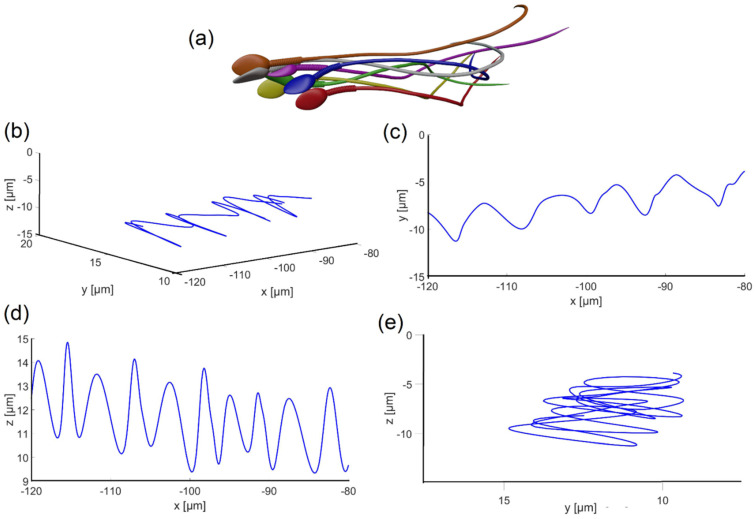
Flagellar beating of the swimming normal model. (**a**) Six frames from the 3D motion of the normal model. (**b**–**e**) Head centroid path in different planes.

In the second case, we dynamically tracked the 3D trajectories of the sperm head centroid of each model across infinite fluid for 17 s, with submicron accuracy. The 3D trajectory of the normal model exhibited the most prevalent swimming pattern reported among healthy human sperm, which is called the “typical” trajectory (>90% of cells) [7,11]. Figure 5a shows six frames (each in a different color) from the flagellar beating of the normal model, as the sperm head changed its direction arbitrarily in 3D space over 16 sec. The path shown in Figure 5b–e indicates that the sperm head moved forward swiftly along a slightly curved axis with a small lateral displacement (approximately 4 μm side-to-side) in either direction orthogonal to the flow, in agreement with earlier observations. The oscillation frequency obtained from the analysis of the waveforms shown in Figure 5 was 5.4 Hz, and the maximum local amplitude was 7.3 µm; in other words, the head model completed 5.4 cycles (rotation of 360° for each cycle) per second with an internal maximum flagellum amplitude of 7.3 µm. Video S5 displays a validation of the flagellar swimming pattern obtained from the dynamic simulation. Here, we verified that the results of the dynamic simulation described virtually the actual swimming pattern of sperm cells, experimentally acquired in our previous study [11].

### 6.2. Normal and Abnormal Model Swimming Patterns

**Figure 6:**
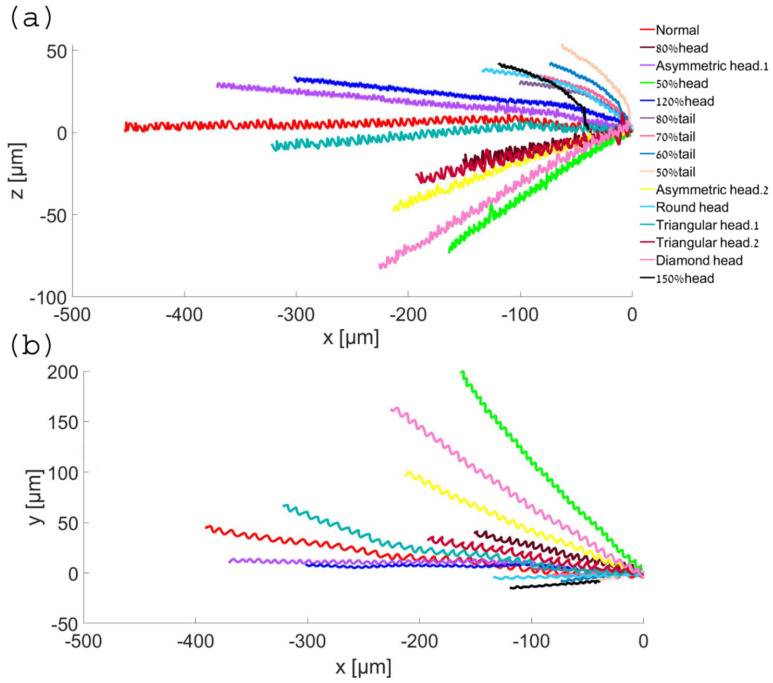
Normal and abnormal model swimming patterns. Two-dimensional trajectories in the forward direction (**a**) and in the upward direction (**b**).

During swimming, we observed that the different sperm models displayed a large variation in frequency, waveform, and path trend in their 3D swimming patterns (Figure 6, see Video S1). The model positions, trajectories, and displacements as a function of time were significantly different. Compared to the normal model, the abnormal sperm models exhibited different 3D swimming patterns, which showed significant 3D lateral displacements, different degrees of periodicity, and twisted trajectories (see Videos S2 and S3).

**Figure 7:**
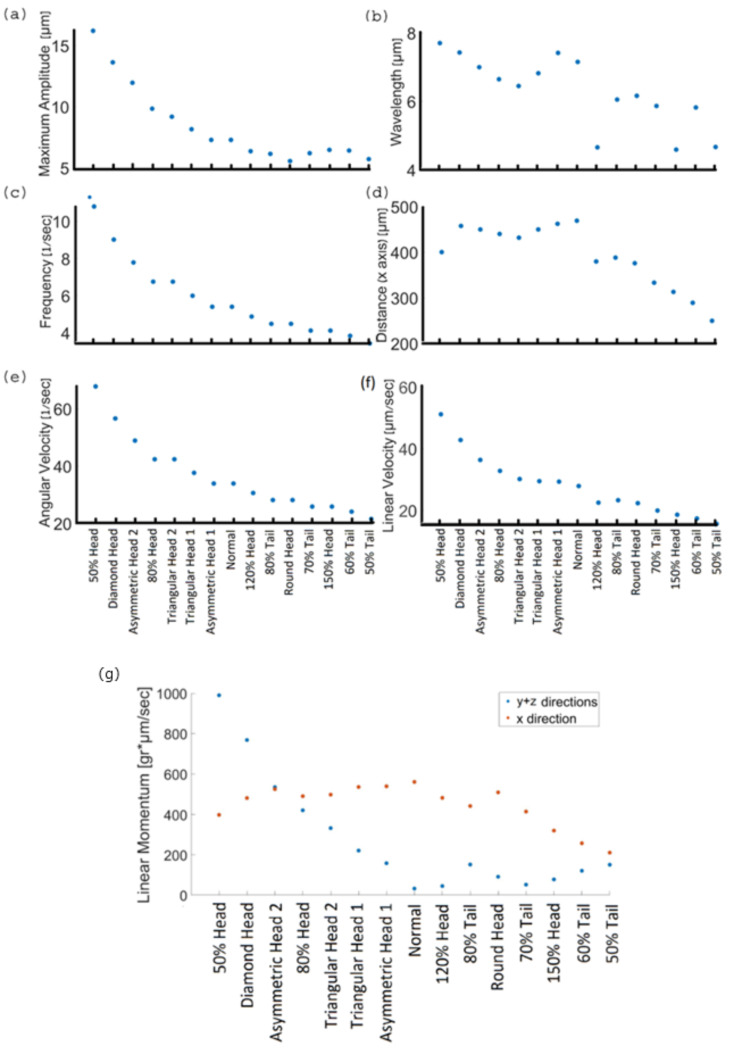
Kinetic parameters of 3D swimming patterns. Maximum amplitude (**a**), wavelength (**b**), frequency (**c**), distance (**d**), angular velocity (**e**), linear velocity, and (**f**) linear momentum values in the forward direction ‘*x*’ (red) and the lateral direction ‘*y* + *z*’ (blue) (**g**) of the different sperm models swimming in saline water.

We also quantified various physical and kinetic parameters of the 3D swimming patterns and compared them to the statistical behavior of human sperm, such as flagellum amplitude, wavelength, beat frequency, total and axial displacements, and linear and angular velocities (Figure 7a–f). The kinetic parameter values of the normal model are equivalent to earlier reports that used different imaging techniques to track healthy human sperm [7,24,38]. Nevertheless, the abnormal models showed a wide range of kinetic and physical values that prevented them from reaching the target.

### 6.3. Kinetic Parameters of 3D Swimming Patterns

The kinetic parameters for the models that had a short tail, large head or rounded head were lower than those of the normal model. For the models with a defective tail, the tail did not have sufficient energy to move forward, so these models swam the shortest distance in the forward direction (332.5 ± 52 µm). The kinetic energy of the normal cell was $E=5.36\;\times \;10^{-8}\;\mathrm{joules}$; each cell with less energy will have difficulty moving itself forward. As a result, their linear and angular velocities were lower, and their motion frequency and wavelengths decreased as well (linear velocity: 20 ± 3 µm/s, angular velocity: 26.3 ± 3 1/s frequency: 4.2 ± 0.5 Hz, wavelength: 5.4 ± 0.72 µm). The internal amplitude also had the lowest values (6.11 ± 0.35 µm) for the same reason that the tail had fewer internal motors due to its short length, which weakened its normal beat. Even the models with a large or rounded head did not get as far from their original location, which means that their frequency, wavelength, and velocity were relatively low compared to the normal model. We can explain this by the load theory, according to which a large head increases the mass of the entire model and offloads the tail. In our case, the load is the total mass of the cell. Thus, these models swam more slowly and for smaller distances compared with the normal model.

On the other hand, the physical values of the 3D trajectories of the models with small, triangular or asymmetrical heads were significantly larger than those of the normal model. Despite the large values of rotational and linear velocity, frequency, internal amplitude, and wavelength (linear velocity: 36 ± 8.25 µm/s, angular velocity: 47 ± 11.8 1/s, frequency: 7.5 ± 1.9 Hz, wavelength: 7 ± 0.5 µm, amplitude: 10.9 ± 3.2 µm), these defected models swam a shorter distance in the forward direction and with a large lateral displacement upwards (441.5 ± 21 µm) compared to the other models. These defected models were inefficient as they deviated from the straight forward swimming path, which could keep them from their goal of reaching the female egg. Moreover, a small head can indicate a deficiency of genetic material in the nucleus, which may cause genetic problems or unsuccessful fertilization. To conclude, the mechanical behavior of the normal sperm model displayed the best 3D swimming pattern for achieving the goal of reaching and fertilizing the egg.

Lastly, we described the mechanical behaviors of the models in terms of momentum; a vector quantity expressing the direction and intensity of the model motions to evaluate the difficulty of the body to change its velocity during free movement by:

(17) $$P_{x,y,z}=m\times v_{x,y,z},$$

where m is the model mass and $v_{x,y,z}$ is its axial velocity.

We described the momentum in two directions, ‘*x*’—the forward direction along the ‘*x*’ axis, and ‘*y* + *z*’—the lateral direction in the ‘*y* − *z*’ plane (Figure 7g). The momentum of the healthy normal model, which was calculated from its dynamic swimming parameters, had the largest value in the forward direction (561 g × µm/s) and the lowest value in the other direction (32 g × µm/s) compared to the abnormal model values. This result indicates that the normal sperm cell applied the most energy towards moving in the forward direction, in addition to a constant velocity in the 3D space. In other words, compared to the abnormal cells, it will be more difficult to interrupt the swimming path of a normal sperm cell by collision with obstacles or with other cells. Compared with the normal model, the defect models had lower momentum values in the forward direction (436 ± 105 g × µm/s), and larger momentum values in the lateral direction (293.8 ± 290 g × µm/s); their kinetic energy was wasted on a different direction, i.e., was not focused in the forward direction, which explains our observations that the 3D sperm swimming patterns showed large lateral displacements and different degrees of periodicities.

### 6.4. Predictions for the Cell Swimming Paths

**Figure 8:**
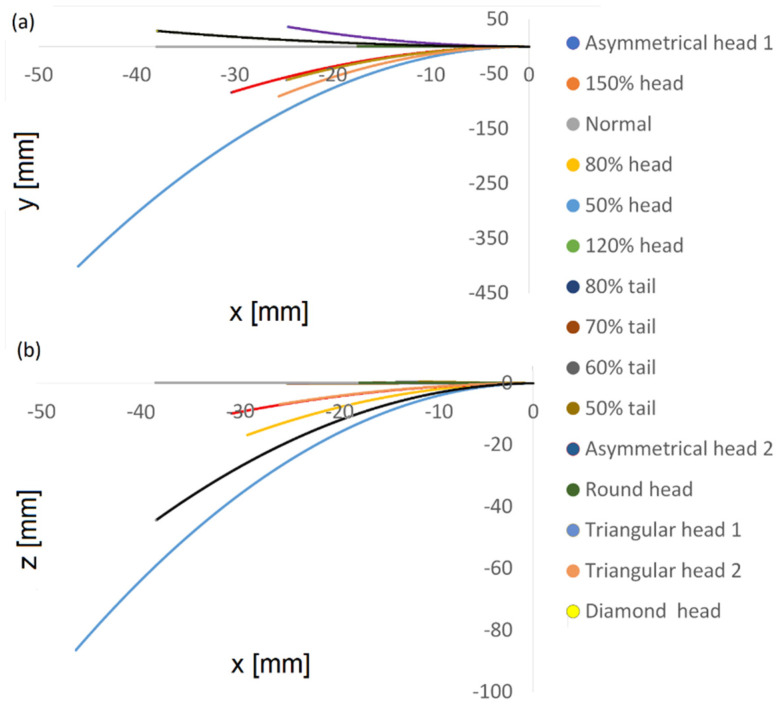
Normal and abnormal model swimming patterns over 15 min, starting on the right. Two-dimensional trajectories in the ‘*x* − *y*’ plane (**a**) and in the ‘*x* − *z*’ plane (**b**).

Based on the dynamic simulation described above, we performed predictions for the cell swimming paths over a longer period to identify global trends and the longer-term behavior of the cells. Using 3D polynomial regression, we identified the relationship between the independent variables, which was modelled as a polynomial in 3D. Based on this, we predicted the paths travelled over 15 min in 3D. Figure 8 shows the two-dimensional trajectories of the normal and abnormal cell models in the ‘*x* − *y*’ plane and in the ‘*x* − *z*’ plane. We found that short-term trends and behavior were preserved over time; the cells kept moving forward and the gaps continued to increase over time between normal and abnormal cells. As shown earlier in the calculated path, the 3D trajectory of the normal model was the most linear along the forward direction, the ‘*x*’ axis, and this held true for the predicted longer path here. Although the abnormal models moved faster or slower in terms of 3D displacement and covered longer distances along the ‘*x*’ axis, the normal model still had the most linear path in this direction, meaning that it was more progressive and conserved more energy. Video S4 shows the 15 cell model paths during the last 5 s of the 15 min swim. The internal oscillation, tail movement, and head rotation of the sperm models were preserved over the entire 15 min duration, and the models continued to progress in the forward direction.

### 6.5. Scoring of Sperm Pathologies

Based on the results described above, we scored the various sperm cell pathologies according to their estimated success rates reaching the egg (Table 1). The kinetic parameters (cell velocity in the forward direction, linearity (deviation angle of the pathological model trajectory from the normal one) and wasted energy (momentum in the lateral direction)), which were calculated by the dynamic simulations (Figure 7), were used to evaluate the sperm cell. The models with a large head had the best chance to reach the egg and the closest behavior to the normal one due to their linear path in the forward direction; nevertheless, their low velocity prevented them from reaching the egg first. Thus, theoretically they would reach the egg in the second place after the normal cell. The sperm models with short tails swam slowly along the forward direction with a deviation that increased with cell shortening, which made them less likely to reach the egg. Triangular and asymmetrical (narrow-side) heads had low chances to reach the egg because of their parabolic trajectories in the forward direction, which could keep them from their goal despite their high velocity. Small, diamond, and asymmetrical (flat side) heads were the faster swimmers due to the significantly smaller mass of the entire model. However, these sperm models deviated from the straightforward swimming path and swam in the lateral direction, with low chances of reaching the egg.

## 7. Discussion

We described the flagellar beats of different human sperm cell models with submicron position resolution and quantified multiple kinetic and physical parameters during free swim. We developed 15 advanced 3D sperm cell models, presenting full sperm geometry containing the actual sperm cell organs (head, flagellum, and midpiece), including internal components. The model geometry was constructed based on our previous experimental rapid optical acquisition method for individual human sperm cells during 3D movement [11].

Full mechanical modeling is a key factor in understanding sperm cell motility and behavior in 3D, which directly impacts the chance of success in fertilization. A more accurate and realistic mechanical model can lead to better identification of the real movement of a swimming sperm cell, leading to improved sperm selection methods. The first model, created according to the experimental 3D video, defined a normal sperm, while the other 14 models defined sperm cells with different types of abnormalities, including known frequent defects in the sperm head and flagellum morphologies. We performed dynamic simulations by activating dynein sliding forces using a mathematical model, characterizing the instantaneous configuration and the forward swimming of the flagellum.

From Video S3, we see that some abnormal sperm cells swim faster than normal sperm cells. This finding is very important for sperm selection in IVF, as sometimes the embryologist selects sperm cells based on their progressive motion only, whereas the sperm morphology cannot be well characterized due to the use of bright-field microscopy. Our findings demonstrate the necessity of imaging sperm cells in IVF with quantitative interferometric computed tomography to characterize the sperm cell morphology as well, and not only progressive motion, in order to avoid the selection of pathologic sperm cells.

We found that the kinetic parameters values of the normal model are equivalent to earlier reports that used different experimental imaging techniques to track healthy human sperm cells [1,7,11,26,28,38]. In addition, we compared the normal sperm model with the abnormal sperm models that have shown a wide range of kinetic and physical values, which ultimately prevent them from reaching the egg. The 3D trajectory of the normal model has the longest path in the forward direction, as the kinetic energy is directed into forward path, without wasting energy in the lateral direction. Our results indicate that the normal model does not move the fastest in terms of 3D displacement. However, previous studies described the sperm speed only in the forward direction and showed a positive correlation between high speed and high fertilization success [38,39,40,41]. Our study can be used to inspect the full mechanical behavior of sperm cells in 3D space in high spatial resolution for a much longer period than is practical experimentally.

Last to be discussed is the rotational behavior of the cells on their progression axis; this rotation grants the cells the ability to bypass obstacles smoothly and is effective resistance against losing a large amount of kinetic energy due to cell collisions. The rotational motion softens the loss of energy in situations where the cell contacts an external body. The fact that the cells have relatively negligible spiral motion relative to their progressive axis rotation emphasizes the mechanism of effectively using the kinetic energy of the cells. This behavior matches the general evolution of the cell motion towards saving its energy for its intended use; to eventually reach the female egg and penetrate it. In the future, incorporation of environmental parameters such as flow, temperature, humidity, and viscosity can advance further study of these parameters and their influence on the behavior of sperm cells. Due to the variety of viscosities and topographies in the fallopian tube, further research and advanced models are needed for predicting the ability of sperm to reach the fertilization area. We intend to expand our research to include barriers and cell-to-cell interactions, including collisions.

This study is the first in the field that predicts cell pathways and classifies cells by pathological groups, performing scoring of the success rates for each cell pathology, and evaluates the chances of reaching the egg according to the kinetic properties and behavior of the cells. Table 1 presents the case of cells swimming in watery medium. Real situations include the true geometry of the female reproductive system, cervical mucus, ciliary movements in the fallopian tubes, etc. In our future studies, we will test improved dynamic simulations, which include sperm-model swimming in cervical mucus (non-Newtonian viscoelastic flow) through the female reproductive system. Thus, this study opens the door to many additional studies in predicting sperm-cell behavior and performing informed selection processes.

To conclude, we have presented new tools for predicting the 3D motion of healthy and pathological human sperm cells over short and long periods of time. Our method is based on a multidisciplinary approach, combining an ultrafast 3D optical stain-free acquisition method and mechanical modeling, as well as prior knowledge on the sperm cell internal structure, extrapolating from short-term 3D optical acquisition of normal human sperm cells to short- and long-term 3D dynamic modeling of both normal and pathological cells. The presented tools are expected to provide missing links from previous studies and may lead to changes in sperm cell analysis and selection methods. Specifically, predicting the long-term 3D motion of normal and abnormal human sperm cells and their mechanical behavior constitutes a novel capability that may help in differentiation and selection strategies of human sperm cells for fertility characterization and treatment, as well as in cell motion research, e.g., biomimetic robotics.

## Figures and Tables

**Table 1:** Scoring of sperm pathology categories according to their chances of reaching the egg.

Category	Defect Description	Cell Behavior (Compared with the Normal Cell)			Conclusion
		Velocity [µm/s]	Linearity (X° from Normal Cell)	Wasted Energy (Momentum in Lateral Direction [g × µm/s])	
Large head	150% head	19 (Low)	Linear (2°)	81 (Medium)	− Best chance to reach the egg due to their linear path. − Not the first ones to reach the egg due to their low velocity. − Large head increases the mass of the entire model and offloads the tail.
	120% head	26 (Low)	Linear (2.2°)	48 (Medium)	
	Round head	25 (Low)	Linear (3.2°)	95 (Medium)	
Short tail	50% tail	19 (Low)	Linear (4.5°)	155 (Medium)	− Good chance to reach the egg due to their linear path, although the deviation is larger from the normal cell. − Not the first ones to reach the egg due to their low velocity. − Fewer internal motors due to their short length, which weakens their normal beat.
	60% tail	20 (Low)	Linear (7°)	124 (Medium)	
	70% tail	23 (Low)	Linear (10°)	55 (Medium)	
	80% tail	26 (Low)	Linear (14°)	155 (Medium)	
Normal (symmetric, 100% head, 100% tail)		31 µm/s	The most linear (0°)	36 gr × µm/s (minimum value)	
Asymmetrical and triangular head	Asymmetrical 1	32 (Medium)	Circular	162 (High)	− Low chance to reach the egg due to their parabolic path that acts as a tendency for escape from their goal. − Slightly smaller head decreases the mass of the entire model; therefore, the tail still pushes less mass in the forward direction.
	Triangular 1	32 (Medium)	Circular	224 (High)	
	Triangular 2	33 (Medium)	Circular	336 (High)	
	Asymmetrical 2	40 (High)	Circular	539 (High)	− Very low chance to reach the egg due to their parabolic path. − Very small head decreases the mass of the entire model; therefore, the tail pushes significantly less mass in the forward direction.
	Diamond	46 (High)	Circular	772 (High)	
Small head	80% head	36 (High)	Circular	424 (High)	
	50% head	54 (High)	Circular	994 (High)	

## Data Availability

All data is available from the corresponding author upon a reasonable request.

## Supplementary Materials

The following supporting information can be downloaded at: https://www.mdpi.com/article/10.3390/cells11081319/s1, Figures S1–S3; Videos S1–S5.
