# Supplementary material for: Prediction of Sperm Progression in Three Dimensions Using Rapid Optical Imaging and Dynamic Mechanical Modeling
Source: Cells. 2022 Apr 13;11(8):1319. doi: 10.3390/cells11081319 (PMC9030059; doi:10.3390/cells11081319)
Supplement: Supplementary file 1 [file cells-11-01319-s001.zip › cells-1625287-supplementary.pdf]

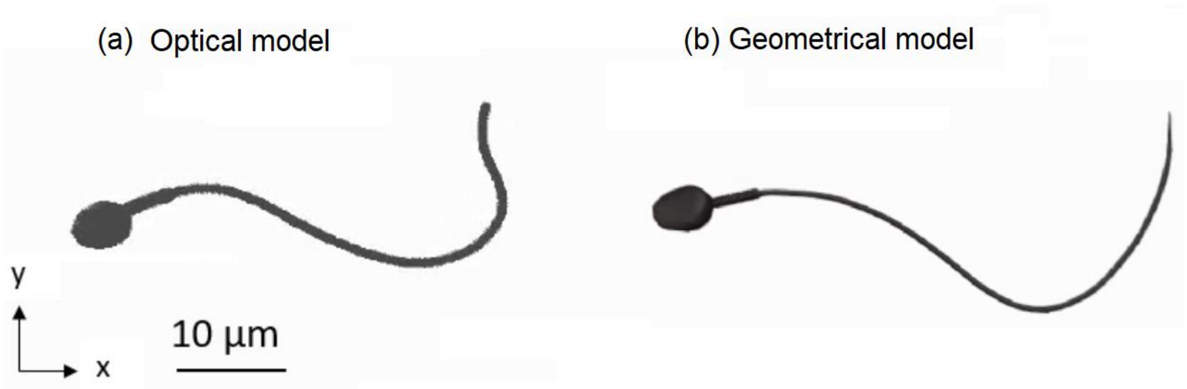

Figure S1. Structural similarity comparison between (a) the optical (experimental), and (b) the geometrical (numerical) models of normal sperm cell. The optical model was obtained from the previous experimental study [10], and the geometrical model has been built in this mechanical study.

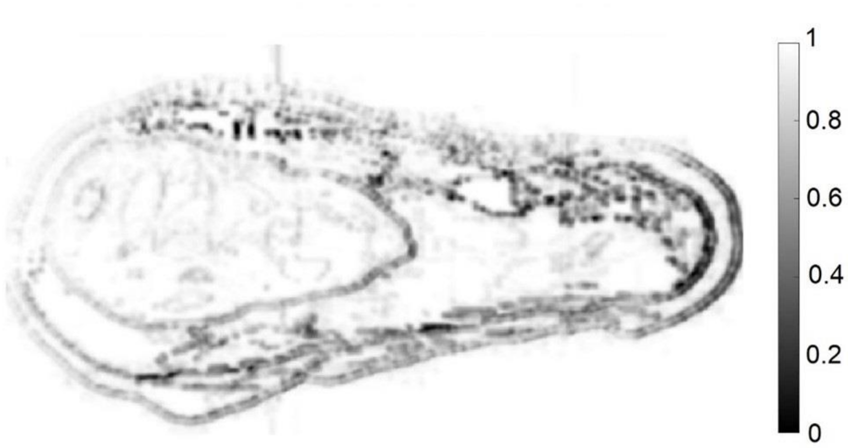

Figure S2. Local structural similarity image index (SSIM) map for the sperm head when comparing the optically acquired healthy sperm cell to our numerical model. SSIM = 0: complete local dissimilarity between the images. SSIM = 1: complete local similarity between the images.

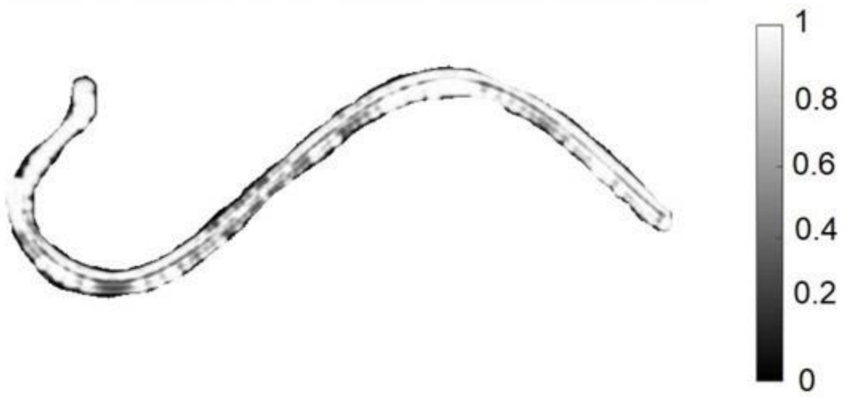

Figure S3. Local structural similarity image index (SSIM) map for the sperm tail when comparing the optically acquired healthy sperm cell to our numerical model. SSIM = 0: complete local dissimilarity between the images. SSIM = 1: complete local similarity between the images.

Videos:

Video 1: Flagellar beating of all sperm models with clamped sperm head.
Video 2: 3D sperm swimming for all sperm models.
Video 3: The projection of the 3D sperm swimming in the x-y plane for all models.
Video 4: Last five seconds out of 15 min total sperm swimming for all models.
Video 5: Normal-morphology sperm cell swimming in fluid.
