# Supplementary figures and images for: Prediction of Sperm Progression in Three Dimensions Using Rapid Optical Imaging and Dynamic Mechanical Modeling
Source: Cells. 2022 Apr 13;11(8):1319. doi: 10.3390/cells11081319 (PMC9030059; doi:10.3390/cells11081319)

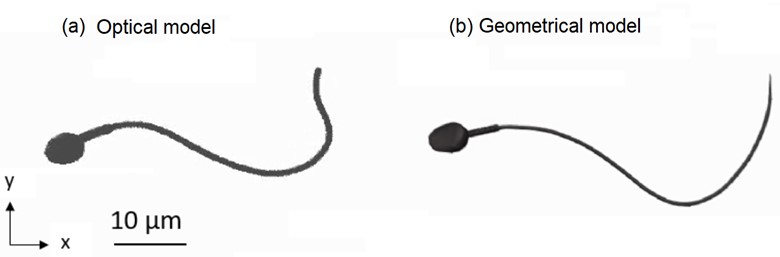

Supplement: Supplementary file 1 [file cells-11-01319-s001.zip › figure S1.jpg]

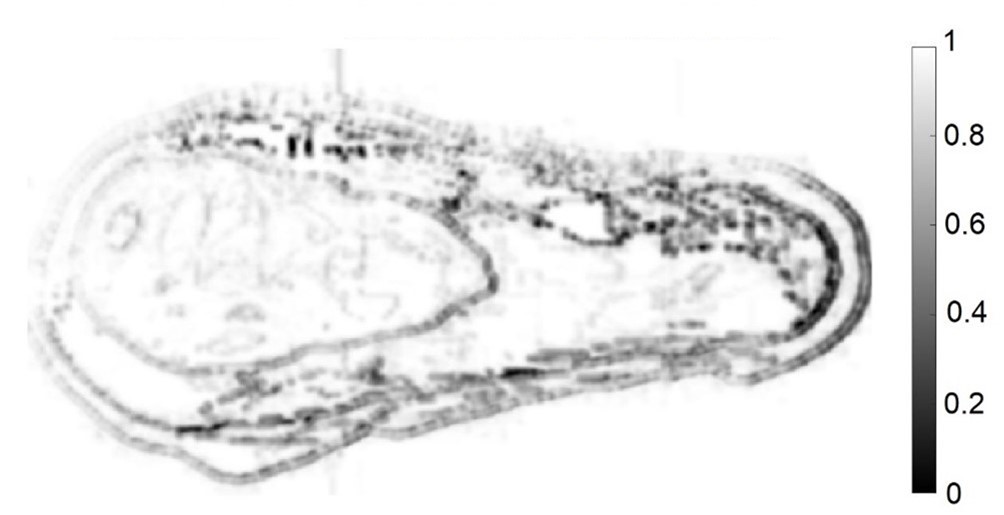

Supplement: Supplementary file 1 [file cells-11-01319-s001.zip › figure S2.jpg]

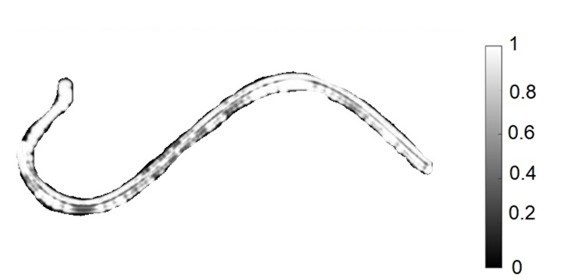

Supplement: Supplementary file 1 [file cells-11-01319-s001.zip › figure S3.jpg]
